# Alexander Mactier Pirrie

**Published:** 1908-01

**Authors:** 


### ALEXANDER MACTIER PIRRIE.

### A MARTYR TO SCIENCE.

A career of great promise has been cut short by the untimely death of Mr. A. Mactier Pirrie. The son of the late Mr. Alexander Pirrie, C. E., he was born on October 2nd, 1882. He obtained his B. Sc. with honours in anthropology at Edinburgh University in 1904, and qualified as M.B.Ch.B., in 1906. He obtained the Carnegie Research Fellowship in anthropology and was appointed anthropologist to the Wellcome Research Laboratories at the Gordon Memorial College, Khartoum. He went out to the Sudan in the autumn of 1906. Under the direction of Dr. Andrew Balfour, the director of the laboratories, Dr. Pirrie made his first expedition up the Nile to the southern limits of the Sudan, and penetrated to remote parts of the Bahr-el-Ghazal. His second expedition took him to the borders of Abyssinia. On both occasions he passed through some of the most pestilential regions of Africa in connection with certain anthropological and physiological researches, appertaining to tropical diseases, upon which the laboratories are engaged. Unfortunately he contracted tropical fever (kala azar), and was so prostrated as to be compelled to return to England, leaving Khartoum on June 17th last. He rallied from the effects of the fever from time to time, but was compelled to enter Chalmers' Hospital, Edinburgh, in October. His death took place on November 12th.He was interred at the Dean Cemetery on November 15th. The Gordon Memorial College, Khartoum, Sir William Turner, Principal and Vice-Chancellor of the Edinburbh University, Mr. Wellcome and others, were represented, and sent wreaths. A resolution of sympathy has been conveyed to the relatives from the trustees of the Gordon Memorial College, and other expressions of sympathy have been received from the Liverpool School of Tropical Medicine, &c &c. It is of interest to note that the first case of kala azar found in Africa, except a case in Tunis referred to by Laveran, was reported by Dr. Sheffield Neave, pathologist to the Wellcome Research Laboratories, Khartoum. Dr. Neave found the Leishman-Donovan body, the parasite of kala azar, in the splenic blood of a patient in the Omdurman Civil Hospital. The discovery is noted by the director in the second report of the laboratories. Dr. Pirrie presented a paper on his African expeditions at the last meeting of the British Association for the Advancement of Science, but was prevented from being present on account of his illness. He brought back a most valuable collection of objects of scientific interest. At intervals during his illness he was engaged on his report to the Carnegie Institute and the Welcome Research Laboratories, Khartoum, for which institutions he acted jointly in the important work he carried out in the Sudan.—*British and Colonial Druggist, Nov. 22, 1907.*

